# Impacts of cigarette smoking on immune responsiveness: Up and down or upside down?

**DOI:** 10.18632/oncotarget.13613

**Published:** 2016-11-25

**Authors:** Feifei Qiu, Chun-Ling Liang, Huazhen Liu, Yu-Qun Zeng, Shaozhen Hou, Song Huang, Xiaoping Lai, Zhenhua Dai

**Affiliations:** ^1^ Section of Immunology, Guangdong Provincial Academy of Chinese Medical Sciences and Guangdong Provincial Hospital of Chinese Medicine, Guangzhou, Guangdong, China; ^2^ Department of Nephrology, The Second Affiliated Hospital of Guangzhou University of Chinese Medicine, Guangzhou, Guangdong, China; ^3^ School of Chinese Materia Medica, Guangzhou University of Chinese Medicine, Guangzhou, Guangdong, China

**Keywords:** cigarette smoking, immunoregulation, adaptive immunity, and innate immunity

## Abstract

Cigarette smoking is associated with numerous diseases and poses a serious challenge to the current healthcare system worldwide. Smoking impacts both innate and adaptive immunity and plays dual roles in regulating immunity by either exacerbation of pathogenic immune responses or attenuation of defensive immunity. Adaptive immune cells affected by smoking mainly include T helper cells (Th1/Th2/Th17), CD4+CD25+ regulatory T cells, CD8+ T cells, B cells and memory T/B lymphocytes while innate immune cells impacted by smoking are mostly DCs, macrophages and NK cells. Complex roles of cigarette smoke have resulted in numerous diseases, including cardiovascular, respiratory and autoimmune diseases, allergies, cancers and transplant rejection etc. Although previous reviews have described the effects of smoking on various diseases and regional immunity associated with specific diseases, a comprehensive and updated review is rarely seen to demonstrate impacts of smoking on general immunity and, especially on major components of immune cells. Here, we aim to systematically and objectively review the influence of smoking on major components of both innate and adaptive immune cells, and summarize cellular and molecular mechanisms underlying effects of cigarette smoking on the immune system. The molecular pathways impacted by cigarette smoking involve NFκB, MAP kinases and histone modification. Further investigations are warranted to understand the exact mechanisms responsible for smoking-mediated immunopathology and to answer lingering questions over why cigarette smoking is always harmful rather than beneficial even though it exerts dual effects on immune responses.

## INTRODUCTION

Cigarette smoking is prevalent worldwide and it has been reported that approximately 1/3 of the adult population smokes tobacco [1]. Smoke from tobacco combustion contains numerous harmful chemicals, including, but not limited to, carbon monoxide, nicotine, nitrogen oxides and cadmium [2, 3]. Exposure of tobacco smoke has been considered as an important cause of preventable death worldwide [4, 5] and related to the development of brain, respiratory, cardiovascular diseases, infections and cancers [6–9] (Table 1). Meanwhile, smoking has been implicated in the production of many immune or inflammatory mediators, including both pro-inflammatory and anti-inflammatory cytokines [10–14]. Recently, many studies have demonstrated that cigarette smoking has far-reaching effects on chronic inflammation and autoimmunity at a systemic level [2, 10, 15, 16], including rheumatoid arthritis (RA), psoriasis, chronic obstructive pulmonary disease (COPD) and systemic lupus erythematosus (SLE). Although reviews have been previously conducted to describe effects of cigarette smoking on various diseases and local immunity associated with a specific disease, a comprehensive review demonstrating impacts of cigarette smoking on major components of immune cells is lacking. We have previously found that smoking hinders long-term allograft survival induced by costimulatory blockade [17]. Here, we aim to systematically review dual influences of smoking on main components of immune cells of both innate and adaptive immunity, and summarize the molecular and cellular mechanisms underlying the effects of cigarette smoking on the immune cells.

**Table 1:** Major diseases caused by cigarette smoking

	Disease		Disease		Disease
Cancers	Lung cancer	Autoimmune diseases	Rheumatoid arthritis	Graft rejection	Cardiovascular graft
	Renal carcinoma		Chronic obstructive pulmonary disease		Renal graft
	Bladder cancer		Systemic lupus erythematosus		Lung transplantaion
	Pancreatic carcinoma		Inflammatory bowel disease		Cardiac transplantation
	Breast cancer		Crohn's disease		Hepatic transplantation
	Hepatocellular cancer		Ulcerative colitis		Lower extremity bypass
	Esophageal squamous cell carcinoma		Psoriatic arthritis		Infrainguinal bypass
	Oral cavity cancer		Ankylosing spondylitis		Skin graft
	Pharynx cancer		Systemic sclerosis		Hematopoietic stem cell transplantation
	Nasopharynx carcinoma		Diabetes mellitus	Oral and respiratory diseases	Acute eosinophilic pneumonia
	Stomach cancer		Macular degeneration		Asthma
	Uterine cervix cancer		Graves' hyperthyroidism		Chronic obstructive pulmonary disease
	Myeloid leukaemia		Goodpasture's syndrome		Hypersensitivity pneumonitis
Pregnancy	Preterm birth		Thromboangiitis obliterans		Rhinitis
	Fetal growth restriction		Primary biliary cirrhosis		Periodontitis
	Placental abrubtion	Neurological diseases	Alzheimer's Disease		Gingivitis
	Placenta previa		Stroke		Recurrent wheezing
	Low birthweight		Small vessel ischemic disease	Cardiovascular diseases	Myocardial infarction
	Sudden infant death syndrome		Cerebral aneurysms		Cardiac arrhythmia
			Silent cerebral infarction		Atherothrombosis
			Parkinson's disease		Thromboangiitis obliterans

## EFFECTS OF CIGARETTE SMOKING ON ADAPTIVE IMMUNITY

### T lymphocytes

**Figure 1:**
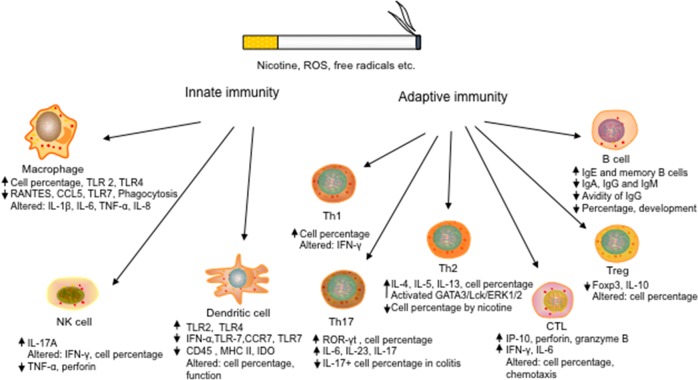
Effects of cigarette smoking on the development and function of both innate and adaptive immune cells Cigarette smoking alters the development, cytokine production, and effector function of both innate immune cells, including DCs, macrophages and NK cells, and adaptive immune cells, such as cytotoxic CD8+ T cells, CD4+ Th cells, regulatory T cells and B cells, leading to pro-inflammatory responses and/or dysfunction of immune cells. (“Altered” denotes contradictory results with both upregulation and downregulation)

T lymphocytes (T cells) are a major subset of immune cells mediating adaptive immunity. In general, activation and differentiation of naive T cells upon antigen recognition generate effector T cells and, at a small frequency, memory and regulatory T cells [18–24]. These cells exert their functions in response to specific antigens through their helper, effector, cytotoxic or regulatory capacities. Previous studies have shown the profound impacts of cigarette smoking on T cells and their release of proinflammatory mediators (Figure 1).

### T helper cells

Epidemiological studies have suggested that either firsthand or secondhand tobacco smoking is an important contributor in the development of many diseases. It's been known that cigarette smoking is a major cause of COPD characterized by chronic airflow obstruction [25]. Forsslund et al [26] analyzed T cells in bronchoalveolar lavage (BAL) fluid and peripheral blood from 40 non-smokers, 40 smokers with normal pulmonary function and 38 COPD patients. They found that the percentage of CD8^+^ BAL cells of smoking groups was higher than that of non-smoking groups while the frequency of CD4^+^ T cells in both BAL and blood of smokers was lower than that of non-smokers. Zhang et el. [27] found that the homeostasis of circulating T helper cells was disrupted in chronic COPD patients compared with healthy non-smokers. Second-hand smoke (SHS) also affected T cell components. Analyses of blood cotinine, a nicotine metabolite, and T-cell subpopulations from non-smokers demonstrated that passive smoking was positively correlated with the prevalence of naive CD3^+^ T cells [28]. Taken together, active smoking increases the percentage of CD8+ T cells but lowers CD4+ T cells in humans while passive smoking generally augments human CD3+ T cells.

Further studies demonstrated that the percentage of Th17 cells in circulating T cell subsets from COPD patients was higher than that of current smokers without COPD and healthy subjects while the percentage of Th1 cells was also increased in COPD patients and current smokers without COPD [29]. Mice with COPD induced by chronic tobacco smoke also exhibited a rise in Th17 subset accompanying with upregulation of Th17-series of cytokines (IL-6, IL-17A and IL-23) in the lung tissue and peripheral blood [30]. A study on BAL from mice, which were exposed to tobacco smoke for at least six months, showed that the number of Th1 and Th17 cells was significantly elevated [31]. Mice with emphysema had an increased expression of Th1-type cytokine IFN-γ and Th17-type IL-17A [32, 33] and/or augmented numbers of Th17/Tc17 and Tc1 cells [33, 34]. Therefore, both murine experiments and human studies suggest that increases in Th1 and Th17 cell subsets are associated with pulmonary inflammation as a result of cigarette smoke exposure (CSE).

Crohn's disease (CD) is a chronic inflammatory bowel disease that leads to obvious morbidity [35], and is epidemiologically correlated with cigarette smoking [36, 37]. Many studies have revealed that immune responses mediated by Th1 and Th17 cells play an important role in CD [38–40], and that nicotine, a major component of tobacco smoke, can worsen the trinitrobenzene sulfonic acid (TNBS)-induced colitis in mice with an increased percentage of Th17 cells [41]. In contrast, CSE was found to have a different effect on Th17 cells in ulcerative colitis. Nicotine relieved oxazolone-induced colitis and reduced the number of Th17 cells in mice [41]. Montbarbon et al pretreated C57BL/6 mice with cigarette smoke for 14 days and then induced their colitis by dextran sodium sulfate (DSS). They observed that smoke exposure improved colonic inflammation with an obviously reduced production of colonic Th1/Th17 cytokines, including TNFα, IFN-γ and IL-17 [42]. The contradictory effects of smoke/nicotine on two types of experimental colitis in mice resulted from different pathologic changes. It has been known that TNBS-induced colitis was Th1 cell-mediated whereas oxazolone-induced colitis was Th2 cell-oriented [43, 44]. Galitovskiy et al. [41] showed that Th1 cytokine IL-12 significantly decreased the protein expression of α7 nicotinic acetylcholine receptors (α7 nAChR), which was expressed on murine CD4+ T cells and relayed anti-inflammatory signals, while Th2 cytokine IL-4 enhanced α7 nAChR expression. Therefore, nicotine exhibited dual effects on colitis of differential animal models due to the opposite expression profile of anti-inflammatory α7 nAChR. CSE also influenced other autoimmune diseases by regulating Th17 responses. Torii et al. evaluated the percentage of circulating Th17 among CD3+ cells in peripheral blood mononuclear cells (PBMCs) of psoriatic patients and found that smokers had higher levels of Th17+ T cells than non-smokers and that tobacco smoke extract enhanced Th17 generation *in vitro* [45]. Moreover, smoking was suggested to induce rheumatoid arthritis by promoting Th17 responses through Aryl hydrocarbon receptor on human T cells [46, 47].

Th2 cells are mainly primed by IL-4 and secrete effector cytokines against extracellular parasites. It was reported that CSE exacerbated the Th2-mediated airway inflammation in mice treated with OVA [48], and enhanced mRNA and protein expression of thymic stromal lymphopoietin [49], which was important for Th2-specific allergic inflammation. It was also observed that prenatal secondhand smoke significantly elevated the secretion of Th2 cytokines, including IL-4 and IL-13, and promoted activation and polarization of Th2 cells and pulmonary inflammation in BALB/C mice [50, 51]. Mishra et al. [52] revealed that nicotine treatments to Brown Norway rats, which were sensitized with allergens, apparently reduced the expression level of pulmonary Th2-related chemokines and cytokines, and inhibited eosinophil migration. These animal studies indicate that cigarette smoking mostly promotes Th2 immune responses as well as Th2-related pulmonary inflammation and asthma, although nicotine may attenuate allergy via reducing Th2 responses.

In summary, data from both human and animal studies indicate that Th17 cell is actively involved in worsening smoking-associated inflammation and autoimmune diseases, including COPD, CD, colitis, RA and psoriasis, although nicotine can mitigate colitis in mice via suppression of IL-17 expression. Moreover, cigarette smoking may promote autoimmune diseases by enhancing Th1 polarization. Smoking also promotes Th2-mediated pulmonary inflammation and allergy in animal studies. Further investigations, especially in humans, are needed to provide mechanistic insight into the effects of cigarette smoke on Th1/Th2/Th17 responses and allergy or autoimmune diseases mediated by these T helper cells.

### CD8^+^ T cells

CD8^+^ T cells are also known as cytotoxic T lymphocytes (CTLs), which play an important role in host immune defense via killing infected or damaged cells. It was reported that chronic CSE could not induce inflammation or immune responses and emphysema in CD8 knockout mice [53]. Further studies demonstrated that IP-10 from CD8^+^ T cells facilitated the production of macrophage elastase, contributing to elastin fragmentation and pulmonary injury [53]. These results indicated that CD8^+^ T cells serve as a key mediator of COPD. Nadigel et al. [54] found that human CD8^+^ T cells, either from lung tissue of COPD patients or exposed to cigarette smoke condensate, expressed more TLR4 and TLR9 proteins as compared with controls, while CSE also induced the activation of circulating CD8+ T cell with an increase in cytokine expression. Moreover, analysis of clinical specimens from 9 smokers with COPD and 7 healthy smokers for lung resection showed that CD8^+^ T cells were also increased in the peripheral airways of COPD patients compared with healthy smokers [55], and their proliferation was induced by CSE [56, 57]. Another study on emphysema mice demonstrated that cigarette smoke not only increased the percentage of IL-21^+^ Th17 and IL-21R^+^ CD8^+^ T cells in peripheral blood, but also enhanced their expressions of IL-17 and IL-21, which in turn upregulated perforin and granzyme B in CD8^+^ T cells, indicating that cytotoxic function of CD8 ^+^ T cells can be regulated by Th17 cells in emphysema [58]. On the contrary, early investigation had revealed that smokers with COPD (n=12) had less circulating CD8^+^ T cells and more chemokine receptor CXCR3 on CD8+ T cells than smokers without COPD (n=14) and nonsmokers (n=13), while smokers with and without COPD had more activated and cytotoxic (CD27-CD45RA+) CD8^+^ T cells in the peripheral blood than normal nonsmokers [59].

In conclusion, overwhelming majority of studies in humans have shown that smoking increases the number of CD8+ T cells and their activation and function. The contradictory data from initial studies showing a reduction in human CD8+ T cell numbers under the smoking condition could be attributed to gender and genetic background or racial difference. However, studies in both humans and animals indicate that cigarette smoke not just alters the total number of CD8+ T cells, but also induces or enhances their functional responses. Meanwhile, these findings suggest that the influences of cigarette smoking on CD8+ T cells may vary, depending on the differential tissue microenvironment and pathological conditions.

### Regulatory T cell (Treg)

Tregs play an essential role in maintaining immunologic homeostasis and tolerance through its immunosuppressive capacity. Epidemiologic investigations have revealed that smoke exposure is associated with the imbalance of Tregs in COPD patients or smokers. Barceló et al. [60] reported a significant downregulation of CD4^+^ CD25^+^ Treg cells in BAL fluid of patients with COPD compared with healthy smokers. Subsequent analyses by other groups demonstrated a similar tendency in circulating CD4^+^ and CD8 ^+^ Tregs of COPD patients [61, 62]. Furthermore, smoking or passive cigarette smoke exposure during gestation contributed to reduced Treg numbers in cord blood [63], resulting in a higher risk of neonatal atopic dermatitis and food allergy. On the other hand, mounting evidence demonstrated that COPD patients had a prominent increase in Treg cells. The analysis of BLA fluid from smokers and COPD patients showed that the percentage of CD4^+^CD25^+^ Tregs was augmented compared with healthy non-smokers [64–66]. Moreover, the prevalence of CD4^+^FoxP3^+^ Treg cells was also elevated in the pulmonary tissue and peripheral blood of COPD patients compared with non-smokers [29, 67]. Although an increased frequency of CD4^+^CD25^+^ T cells was observed in smokers with normal pulmonary function, the alteration of FoxP3^+^ and CD127^+^ expression was not seen when compared to non-smokers [66]. Three subpopulations of human Tregs were reported. The suppressive subpopulations contained both resting CD25^++^CD45RA^+^ Tregs (rTregs) and activated CD25^+++^CD45RA^−^ Tregs (aTregs) while the pro-inflammatory subpopulations were cytokine-secreting CD25^++^CD45RA^−^ (FrIII) cells [68]. Hou et al [69] found that COPD patients had a lower percentage of suppressive Tregs (rTregs and aTregs) but higher percentage of FrIII cells compared with healthy smokers, although the frequencies of three subsets of Tregs were all increased in smokers compared to non-smokers, suggesting that Treg imbalance (aTreg+rTreg vs. Fr III) has an impact on pathogenesis of COPD.

Taken together, impacts of cigarette smoking on human Treg numbers remain contradictory. We propose that cigarette smoking impairs immunosuppressive function of Tregs by reducing the number of suppressive Tregs or increasing the prevalence of non-suppressive Tregs, leading to an enhanced autoimmune component in COPD pathogenesis, while increased Treg numbers may occur in some smokers under circumstances, leading to worsened respiratory infections. More in-depth studies are required to clearly define net impacts of cigarette smoking on Treg generation and function in smokers with or without a specific medical condition.

### B cells

Recent investigations have focused on the mechanisms underlying smoking-induced changes in distribution and function of B cells. Epidemiologic studies showed that cigarette smoking resulted in higher prevalence of (class-switched) memory B cells in peripheral blood and memory IgG+ B cells in the lung [70, 71]. Smokers also exhibited an elevated level of circulating IgE, leading to the potential development of atopic diseases and asthma [72]. It has been reported that nicotinic receptors, including alpha4 and alpha7 subunits, are present and play important roles in B cell lines [73, 74]. Chronic nicotine exposure increased the expression of alpha4 and alpha7 subunits and induced proliferation of hybridoma B cells [73]. A retrospective study on prostate inflammation showed that the risk of acute inflammation of current smokers was higher than that of former smokers (OR, 1.35; P, 0.001) and never-smokers (OR, 1.36; P, 0.001), and the risk of chronic inflammation in the baseline biopsy was related with current smoking, indicating that cigarette smoking was correlated with acute and chronic prostatic inflammation [75]. Cigarette smoking also caused inflammation in prostate cancer and a B cell signature in prostate tumors in current smokers, contributing to an increase in the expression of immunoglobulin by B cells infiltrating the tumor [76]. On the other hand, smokers with Helicobacter pylori (H. pylori) infection had a lower number and impaired function of regulatory B cells than non-smokers with also H. pylori infection [77]. Moreover, analyses of immunoglobulins demonstrated a decreased production of IgA, IgG and IgM in smokers [78–80] while a study on the avidity of IgG using modified VLP ELISAs revealed that the higher risk of having the low avidity of HPV16/18 IgG in B cells was also associated with cigarette smoking [81]. Recent investigations have focused on the mechanisms underlying smoking-induced distribution and development of B cells. They developed from bone marrow-derived hematopoietic stem cells that first differentiated into precursor and progenitor B cells and then immature B cells [82]. It was found that tobacco smoke exposure led to obvious downregulation of murine marrow B220^+^CD34^−^ pre-B cells and/or B220^+^CD34^+^ pro-B cells without significant changes in cell apoptosis and cell cycle [83, 84].

In summary, studies on humans again have generated contradictory data showing that cigarette smoking increases frequency of memory B cells and IgE production, lowers regulatory B cell numbers, but decreases production of IgA, IgG and IgM in smokers while smoke exposure downregulates murine marrow pre-B cells or pro-B cells. Meanwhile, smoking raises the risk of inflammation in prostate cancer and B cell signature in the tumors.

### Memory lymphocytes

Memory T cells are a subset of T lymphocytes that have been previously challenged by foreign pathogens or antigens and can respond rapidly and vigorously upon reencounter with the same antigen [85]. Similarly, memory B cells can quickly and effectively generate antibodies upon encounter with a previously-met antigen [86]. Thus, both memory lymphocytes play important roles in human immune defenses. Early studies showed that tobacco smoking apparently elevated memory T cells (CD3^+^CD45RO^+^, CD4^+^CD45RO^+^) and class-switched memory B cells in human peripheral blood [70, 87–89]. Active smoking in COPD patients also induced high levels of class-switched memory B cells in blood and IgG+ memory B cells in the lung [71]. However, subsequent findings indicated an opposite effect of tobacco smoking on human memory T cells. Vardavas et al [28] found a significant correlation of secondhand smoke with reduced frequencies of CD3^+^CD45RO^+^ and CD4^+^CD45RO^+^ memory T cells in the blood of children, accompanying with augmented percentages of CD3^+^ and CD4^+^CD45RA^+^ naive T cells. We speculate that the contradictory roles of cigarette smoke in the circulating memory T cells of adults and children are possibly due to immature immune system in children, which is different from that of adult immune system. Cigarette smoking seemed to attenuate rather than strengthening the response of children memory T cells via suppressing their generation.

Secondhand smoke exposure reduced effector and memory T cells in the lungs and spleens of mice infected with Mycobacterium tuberculosis [90], demonstrating suppressive effects of cigarette smoke on immune responses to infection. Further investigations showed that *in vitro* pretreatments with 4-(Methylnitrosamino)-1-(3-pyridyl)-1-butanone (NNK), a major carcinogen component of tobacco, impaired the expansion of cytotoxic T lymphocytes (CTLs) following their transfer into mice but elevated the frequency of precursor memory CLTs, resulting in a final moderate decline in memory CLTs [91]. Moreover, acute nicotine exposure attenuated the expansion of murine CTLs *in vivo* after transfer as well as their later differentiation into memory CTLs [92].

In summary, smoking enhances T cell memory in adult while reducing it in children. In mice, smoking also reduces memory T cells, especially CTLs. These results indicate that cigarette smoking exerts duel influences on the generation of memory T cells, perhaps depending on an individual's genetic background and environment.

## EFFECTS OF CIGARETTE SMOKING ON INNATE IMMUNITY

Growing evidence has indicated the positive association of cigarette smoking with abnormality of innate immune responses [93–95] although the potential mechanisms are still poorly understood. Kearley et al. [96] found that cigarette smoke exposure (CSE) elevated the IL-33 release from epithelial cells and altered the expression of IL-33 cognate receptor ST2 in different immune cells. They found that smoke exposure enhanced ST2 expression by macrophages and NK cells, but diminished it in group 2 innate lymphoid cells (ILC2s), contributing to strengthened IL-33-dependent pro-inflammatory responses of macrophages and NK cells upon infections. These results indicate complicated influences of smoking on innate immune system. Innate immune cells, including dendritic cells (DCs), natural killer (NK) cells and macrophages etc., play important roles in the host defense against infections. Effects of cigarette smoking on the innate immune cells (Figure 1) are described below.

### Smoking and toll-like receptors (TLRs)

TLRs are a class of proteins that play an essential role in the innate immune system. They are single and non-catalytic receptors generally expressed in innate immune cells, including macrophages and dendritic cells, and recognize structurally conserved molecules that are derived from pathogens. Botelho et al. found that CSE resulted in inflammatory responses mediated by neutrophils and monocytes, while activated CD4+ T cells were presented in murine lungs after the prolonged exposure, implying that innate immune cells are sufficient to trigger the acute inflammation in a response to smoke stimulation [97]. The acute inflammatory responses caused by smoking was reported to depend on toll-like receptors (TLRs) [98]. Furthermore, Doz and colleagues showed that cigarette smoking (with two cigarettes twice in a day for three days) caused acute airway inflammation in mice through TLR-4 and IL-1R1 signaling [99]. Cigarette smoking also promoted inflammatory responses and atherosclerosis by activating the H1R-TLR2/4-COX2 axis [100]. Study on patients with periodontitis revealed that smoking enhanced the mRNA expression of TLR-2 and TLR-4 in the gingival tissue [101, 102]. Similarly, increased expression of TLR-2 was observed in the lungs of mice exposed to cigarette smoke [103]. These results indicate that cigarette smoking induces inflammation via increasing the expression and responsiveness of TLRs. On the other hand, it was revealed that maternal smoking reduced the TLRs (TLR-2, TLR-3, TLR-4 and TLR-9) responsiveness of infants' cord blood cells compared with nonsmoking groups, possibly increasing the risk of respiratory infections and asthma [104]. And CSE caused a decrease in mRNA level of TLR-7 and IRF-7 in human plasmacytoid DCs (pDC) infected by respiratory syncytial viruses, demonstrating a suppressive effect of cigarette smoke on pDC upon infection [105]. Taken together, cigarette smoking is likely to exacerbate inflammatory responses but attenuate immune defenses against infections by regulating TLR signaling.

### Dendritic cells (DCs)

DCs are derived from a hematopoietic lineage of bone narrow and can induce immune responses to pathogens via processing and presenting antigens [106]. Cigarette smoke alters the number, distribution and development of DCs and Langerhans cells (LCs). It was reported that active smoking correlated with augmented numbers of DC/LC lineage and caused a dramatic increase in the number of LCs in human alveolar parenchyma [95]. And cigarette smoking upregulated the expression of CCR7, MHCII and CD86, and significantly promoted the trafficking and responses of airway DCs in mice sensitized with OVA, facilitating the allergic airway inflammation [107]. Moreover, passive smoking enhanced the frequency of murine pulmonary DCs and caused their accumulation and activation, which relied on IL-1R1/IL-1α [108]. The upregulation of DC numbers in individuals exposed to cigarette smoke likely resulted from a rise in the cell survival, which was supported by a previous study on the responsiveness of human and murine DCs to smoke exposure [109]. Thus, smoking possibly aggravates the airway inflammation through increasing both the number and function of DCs in humans as well as mice.

Mounting evidence has indicated that cigarette smoke or its extract also negatively regulates the function and maturation of DCs. It was demonstrated that CSE was significantly associated with the reduced stimulating capacity of DCs in mice with asthma [110], and that murine DCs treated *in vitro* with carbon monoxide (CO), a component of tobacco smoke, prevented accumulation of pancreatic autoreactive CD8^+^ T cells in mice with autoimmune diabetes [111]. Furthermore, CSE led to the reduced pulmonary DCs and suppression of DC maturation in murine lymph nodes, accompanying with the decreased expression of MHC II and costimulatory molecules (CD80 and CD86) and an attenuated capacity of inducing T cell proliferation [112]. The smoke exposure for a longer than 24 hours resulted in suppression of functional development of DCs with downregulation of MHCII, CD83, CD86 and CD40, as well as a decline in CD45 expression on human DC cell line L428 [113]. Similar effects of cigarette smoke were reported in human studies. The prevalence of mature DCs (CD83^+^) and migratory DCs (CCR7^+^) was decreased while the percentage of immature DCs (CD1a^+^) was obviously increased in the lung tissues of COPD patients compared with healthy non-smokers [114]. Moreover, smokers with COPD had lower mRNA expression of CD83 and CCR7 than healthy non-smokers [114]. Plasmacytoid DCs were present in tissues that were in close association with the external environment and important for immune defenses against viruses [115]. Cigarette smoke extract was shown to reduce the expression of IFN-α and TLR-7 in pDC from healthy human volunteers and in pDC infected by respiratory syncytial virus, indicating that smoking attenuates the antiviral function of pDC [116, 117].

In conclusion, cigarette smoking profoundly impacts the development and function of DCs and, hence, inflammation. However, findings concerning the impacts of cigarette smoke on DCs are contradictory given that smoking can either suppress or promote DC development and function in both humans and mice. It has not been well defined likely due to the complex compositions of cigarette smoke, the exposure time and quantity of smoke and the interactions between DCs and other immune cells in animal models and humans. Further investigations are necessary to determine the exact effects of smoking on DC generation and function in a specific disease setting and at a particular location.

### NK cells

NK cells are similar to cytotoxic lymphocytes expressing perforin, granzymes, TNF-α and IFN-γ [118], and are a critical component of innate immune cells. NK cells can rapidly and effectively respond because they also exhibit a memory feature [119]. Motz et al. [120] assessed the influences of smoking on NK (CD335+) cells in COPD mice and revealed that smoke exposure promoted the expression of IFN-γ and CD107a in NK cells upon stimulation, and enhanced NK cell responses. Murine NK cells were also primed by cigarette smoke to express more Th-17 cytokine IL-17A [121]. Meanwhile, CSE for over four days activated CD69+ NK cells in murine lung and induced their responses [128]. Further analysis of human data showed that smokers with or without COPD had an increase in the frequency of circulating NK (CD56+CD3-) cells compared with former smokers with COPD and healthy nonsmokers [122, 123]. On the other hand, previous investigations also demonstrated an obvious reduction of NK (CD16+) cells in the peripheral blood of healthy smokers and the smokers exposed occupationally to organic solvents compared with nonsmokers [124, 125]. Cigarette smoke was reported to suppress the expression of IFN-γ and TNF-α in human NK (CD56+CD3-) cells stimulated by poly I:C while smoking-conditioned medium (SCM) reduced the cytotoxicity of NK cells that had a lower perforin production [126, 127]. Similarly, Mian et al. found that cigarette smoke apparently attenuated the activation and cytolytic capacity of human NK cells with decreased expression of activation marker CD69 [128].

Taken together, smoking still exerts dual effects on the frequency and function of NK cells in both mice and humans. The actual influences of cigarette smoking on NK cells may vary, depending on the differential pathological conditions or disease settings and subsets of NK cells with different surface markers. Different subsets of NK cells may paradoxically respond to cigarette smoke in a given setting at a given time.

### Macrophages

Macrophages respond to exogenous pathogens via phagocytosis and digestion, and recruit/activate lymphocytes via their antigen-presenting ability [129]. Ko and others reported that both smoking and nicotine treatments could enhance the expression of proinflammatory chemokine IL-8 in macrophages of both humans and mice [130–132]. Metcalfe et al [133] found that cigarette smoke extract inhibited the responses of COPD-derived alveolar macrophages to TLR signaling and Haemophilus influenza stimulation. These results indicated that smoke-treated human macrophages and IL-8 produced by these macrophages facilitated inflammation, although studies on murine macrophages demonstrated that smoking remarkably suppressed the phagocytosis of macrophages and enhanced bacterial survival [134]. Another report showed a similar trend in phagocytic function of human macrophages THP-1 treated with cigarette smoke extract [135], with an increase in M2 macrophages. M2 macrophage is regarded as a subset of anti-inflammatory cells that can attenuate inflammation, whereas M1 macrophage is referred to as pro-inflammatory cells [136]. Finally, it was found that bone marrow-derived mast cells exposed to cigarette smoke promoted the polarization of murine macrophages into M2 subset [137].

In summary, smoke treatments stimulate human macrophages to release IL-8, facilitating inflammation rather than directly enhancing their function while cigarette smoking suppresses the phagocytosis of murine macrophages. However, smoking promotes M2 polarization of both human and murine macrophages. Further studies are needed to fully understand impacts of smoking on the function of macrophages, especially in humans.

## MOLECULAR MECHANISMS UNDERLYING SMOKING-ASSOCIATED IMMUNOPATHOLOGY

Cigarette smoke is an important source of hazardous chemicals, including nicotine, reactive nitrogen species (RNS), reactive oxygen species (ROS), free radicals, nicotine and polycyclic aromatic hydrocarbons. They cause oxidative stress, DNA damage, inflammation and various cancers [3, 138, 139]. The molecular mechanisms behind the smoking-induced effects on immune cells are still poorly understood. Early investigation revealed that cigarette smoke initiated the MAPK signaling pathways, which in turn regulated the activation of transcription factors (TFs) and affected DNA-binding capacity of more than 20 TFs, including nuclear factor-kappa B (NFκB) [140]. The functional alterations of TFs contributed to transcriptional changes of their target genes, including inflammatory cytokines and chemokines. Furthermore, nicotine was also reported to exert anti-inflammatory effects on activated immune cells via nicotinic acetylcholine receptors (nAChRs) mediated molecular pathways. Nevertheless, the exact molecular mechanisms underlying smoking-associated immunopathology remain largely unknown.

### NFκB

Activation of NFκB with oxidative stress plays a key role in inflammation [141]. It was reported that cigarette smoke induced degradation of IκB-α and activation of nuclear factor-kappa B (NFκB) in lymphocytes and other types of cells, resulting in increased expression of cyclooxygenase-2 and iNOS [142, 143]. An analysis using protein/DNA array showed that CSE strengthened the transcriptional activity of NFκB via promoting its nuclear translocation and DNA binding activity in human A549 cells [140]. Lerner et al [144] demonstrated that cigarette smoke facilitated the expression of cytokine IL-8 and attenuated differentiation of human monocytes via activating NFκB pathway. Furthermore, Reynolds and colleagues found that CSE enhanced the activation of Ras and NFκB, and that downregulation of the receptor for advanced glycation end-product (RAGE) resulted in the reduced activation of NFκB in alveolar epithelial cells [145]. Thus, it was suggested that cigarette smoke stimulated alveolar epithelial cells to express more cytokine IL-1β and chemokine CCL5 via RAGE-mediated Ras-NFκB pathway, possibly contributing to leukocyte recruitments. On the contrary, others demonstrated that CSE suppressed the activation of NFκB in human and murine tracheobronchial epithelial cells infected by Haemophilus influenza (H. influenza), and these findings were supported by a study using animals infected with H. influenza [146]. Mian et al. also observed that smoke-conditioned media significantly suppressed the activation of NFκB and IRF-3 in nonsmokers' PBMCs treated with poly I:C [147], while cigarette smoke extract was shown to dramatically elevate the DNA binding activity of AP-1 rather than NFκB in endothelial cells of human umbilical core vein [148].

Taken together, previous studies indicate smoking also exerts dual roles in regulating NFκB activation in both humans and animals. The net effects of cigarette smoking on NFκB activity differ widely, depending on cell types and extracellular environment with or without exogenous pathogens, which possibly contributes to a decline in immunity against bacterial infections but an increase in pulmonary inflammation.

### ERK

There are three major types of MAP Kinase pathways, including ERK1/2, JNK/SAPK and p38 pathways [149]. Iles et al. found that 4-hydroxynonenal (HNE) induced by cigarette smoke in pulmonary epithelial cells enhanced the phosphorylation of ERK, JNK and c-Jun and the binding capacity of AP-1 with upregulation of Heme oxygenase-1 (HO-1) [150]. Similar ERK-c-Jun pathway induced by CSE was reported by others. Li et al. [151] revealed that CSE induced ERK phosphorylation, which in turn phosphorylated c-Jun in smooth muscle cells, contributing to cyclin D1 upregulation. They also demonstrated the involvement of MEK/ERK1/2 MAPK pathway in the diminished expression of cystic fibrosis transmembrane conductance regulator (CFTR) induced by cigarette smoke in human bronchial epithelial cells [152]. In addition to acting on epithelial cells and smooth muscle cells, CSE treatments also enhanced ERK phosphorylation and suppressed IL-12p70 expression in mature DCs, while the ERK phosphorylation in turn increased nuclear TF c-Fos, leading to the reduction in IL-23 protein levels [153]. It remains to be defined whether cigarette smoke affects ERK phosphorylation in adaptive immune cells.

### P38 MAPK

Both *in vitro* and animal studies have shown that cigarette smoke exposure (CSE) exerts its effects through p38 MAPK signaling pathway. It was reported that CSE apparently elevated the phosphorylation of p38 MAPK in mice with smoke-induced pulmonary inflammation [154, 155]. Furthermore, Moretto et al. [156] found that CSE enhanced both mRNA and protein expression of IL-8, which was important for neutrophil chemotaxis, accompanying with phosphorylation of p38 MAPK and MEK2 in human pulmonary cells. Treatments with inhibitors of p38 MAPK or MEK2 accelerated the degradation of IL-8 mRNA. Thus, they suggested that cigarette smoking augments IL-8 expression in pulmonary structural cells through p38 MAPK/MEK pathway, resulting in neutrophil recruitments into the lungs and inflammatory sites. Additionally, some investigations [157, 158] demonstrated that both p38 MAPK and ERK1/2 pathways were concurrently implicated in the secretion of IL-8 and pulmonary inflammation induced by cigarette smoking.

In conclusion, tobacco smoking activates MAPK signaling in both murine and human pulmonary resident cells and leukocytes, and hence induces the expression of proinflammatory cytokines such as IL-8.

### Histone modification

In addition to effects on NFκB and MAPK signaling pathways, tobacco smoke also alters the cellular chromatin via histone modification [159]. Previous studies established an association of tobacco smoking with augmented acetylation of histone 4 and phosphorylated-histone 3 in human and mice [154, 160]. Yang et al. [161] revealed that CSE attenuated the activity of histone deacetylase (HDAC) and reduced the production of HDAC1, HDAC2, and HDAC3 in human macrophages. Furthermore, expression of SIRT1, a type of histone/protein deacetylases [162], was suppressed by cigarette smoke in inflammatory cells of murine lungs as well as macrophage cell lines, resulting in abrogation of the interaction of SIRT1 with RelA/p65 and acceleration of RelA/p65 acetylation [163]. Since chromatin structures regulated by histone acetylation and deacetylation affected gene transcriptions [164], smoke-induced alterations in histone modification could lead to aberrant gene transcriptions in various immune cells. Taken together, smoking alters the cellular chromatin of both murine and human macrophages via histone modification.

### Impacts of nicotine on molecular signaling pathways

Nicotine has been shown to be an immunosuppressive agent that can modulate innate and adaptive immune responses [165, 166] through interacting with nAChRs on the surface of immune cells, including macrophages, T and B lymphocytes [167]. Recently, considerable work has been done to show that α7 nAChR, one type of nAChRs, plays a crucial role in nicotine's anti-inflammatory effects. The activation of α7 nAChR by nicotine in murine macrophages interacted with Jak2 and then induced the phosphorylation of STAT3, which subsequently inhibited the transcription of pro-inflammatory cytokines [168]. Furthermore, activated α7 nAChR suppressed the phosphorylation of IκB in human monocytes, resulting in inhibition of nuclear translocation of NFκB [166, 169]. Besides, nicotine may regulate additional signaling pathways beyond activation of nAChRs. Early studies showed that nicotine facilitated the release of alpha-melanocyte-stimulating hormone (alpha-MSH) in frog melanotrophs through inducing inositolphospholipid breakdown and increasing the intracellular Ca(2+) concentration, indicating the involvement of non-cholinergic nicotine receptor in nicotine mediated effects [170]. It was also reported that nicotine treatment enhanced Ca(2+) channels and suppressed nitric oxide (NO) signaling pathways in smooth muscle cells of rats [171]. Moreover, interleukin-1 receptor-associated kinase M (IRAK-M), a negative regulator of innate TLR-mediated immunity, was involved in the anti-inflammatory effects of nicotine through α7 nicotinic receptor in human macrophages [172]. Although the major evidence has revealed that nicotine functions via both nAChRs and non-nAChRs in immune cells, the exact signaling pathways of nicotine are still largely unclear and more studies are required to fully explore its molecular mechanisms.

## CONCLUSION

Ample evidence has shown that both innate immunity and adaptive immunity are susceptible to cigarette smoke, which interrupts immunological homeostasis, causes various diseases, and exerts paradoxical effects on immune and tissue cells through regulating NFκB and MAPK signaling as well as histone modification. In particular, cigarette smoke acts as a double-edged sword that either exacerbates pathological immune responses or attenuates the normal defensive function of the immune system, possibly owing to the complexities and functional diversities of cigarette smoke components and individuals' medical condition. Nevertheless, smoking plays a harmful rather than beneficial role in either case. Perhaps, tobacco smoke manufactured from different parts of the country may differ in actual chemical components. It is unknown why smoking is always deleterious rather than beneficial, even though it exerts dual effects on immune responses. For instance, cigarette smoke generally weakens immunity against infections but paradoxically promotes autoimmunity. We speculate that the weakened immunity with prolonged chronic infection results in cross-reactive autoimmunity against both a pathogen and cross-reactive self-tissue. It is also possible that cigarette smoke exerts differential effects on immunity in the context of various regional immunopathology and diseases. Although previous studies have revealed some of the cellular and molecular mechanisms responsible for immunoregulation induced by cigarette smoke, the exact mechanisms underlying smoking-associated immunopathology remain mostly unclear, which warrants further investigations.
